# An Instagram-Based Study to Understand Betel Nut Use Culture in Micronesia: Exploratory Content Analysis

**DOI:** 10.2196/13954

**Published:** 2020-07-09

**Authors:** Wayne Buente, Francis Dalisay, Pallav Pokhrel, Hanae Kurihara Kramer, Ian Pagano

**Affiliations:** 1 School of Communications University of Hawaii at Manoa Honolulu, HI United States; 2 Communication and Fine Arts Division College of Liberal Arts & Social Sciences University of Guam Mangilao Guam; 3 Population Sciences in the Pacific Program (Cancer Prevention in the Pacific) University of Hawaii Cancer Center University of Hawaii at Manoa Honolulu, HI United States

**Keywords:** betel nut, areca catechu, areca, cancer, health, Guam, Micronesia, Instagram, mobile phone, culture

## Abstract

**Background:**

A 2012 World Health Organization report recognizes betel nut use as an urgent public health threat faced by the Western Pacific region. However, compared with other addictive substances, little is known about how betel nuts are depicted on social media platforms. In particular, image-based social media platforms can be powerful tools for health communication. Studying the content of substance use on visual social media may provide valuable insights into public health interventions.

**Objective:**

This study aimed to explore and document the ways that betel nut is portrayed on the photo-sharing site Instagram. The analysis focuses on the hashtag #pugua, which refers to the local term for betel nut in Guam and other parts of Micronesia.

**Methods:**

An exploratory content analysis of 242 Instagram posts tagged #pugua was conducted based on previous research on substance use and Instagram and betel nut practices in Micronesia. In addition, the study examined the social engagement of betel nut content on the image-based platform.

**Results:**

The study findings revealed content themes referencing the betel nut or betel nut tree, betel nut preparation practices, and the unique social and cultural context surrounding betel nut activity in Guam and Micronesia. In addition, certain practices and cultural themes encouraged social engagement on Instagram.

**Conclusions:**

The findings from this study emphasize the cultural relevance of betel nut use in Micronesia. These findings provide a basis for empirically testing hypotheses related to the etiological roles of cultural identity and pride in shaping betel nut use behavior among Micronesians, particularly youths and young adults. Such research is likely to inform the development of culturally relevant betel nut prevention and cessation programs.

## Introduction

### Background

Betel or areca nut is the fruit of a palm tree (*Areca catechu*) consumed as a psychostimulant by over 600 million people worldwide [1]. Betel nut chewing stretches from the African continent to the islands of Micronesia and has been prevalent since the dawn of recorded time [2]. Betel nut is enjoyed by both men and women as well as the young and old, often in combination with tobacco. Similar to other psychostimulants, betel nut chewing is known to cause a sense of euphoria, well-being, feelings of warmth, and increased capacity to work [3]. However, there are a number of health issues associated with betel nut consumption, most notably oral cancer [4].

Betel nut use is best understood as an orphan disease in the medical sense. It is consumed by non-Europeans in poor countries and under researched both in the West and in countries where it is endemic [5]. Increasingly, governments and health organizations are viewing betel nut chewing as a preventable threat to public health and a drain on medical resources.

In 2004, the International Agency for Research on Cancer classified the use of betel nut, with or without tobacco, as carcinogenic to human beings [6]. A 2012 World Health Organization report recognizes betel nut use as an urgent public health threat faced by the Western Pacific region, including Micronesia. Micronesia includes US territories such as the Northern Mariana Islands and Guam and island nations in free association with the United States, such as the Republic of Palau, Federated States of Micronesia, and the Republic of the Marshall Islands [7]. Cancer is 1 of the 2 leading causes of mortality in the US-Affiliated Pacific Islands (USAPI) [8,9]. Lung and oral cancer incidence and mortality rates are markedly higher in the USAPI than in the mainland United States [10].

Scholars have begun examining how social media platforms such as Facebook, Twitter, and YouTube contribute to alcohol use [11-13], smoking [14,15], and other health concerns [16,17]. Social media use in public health communication has been characterized as still in a *wild west* phase, where practitioners design and employ untested strategies [18], and the role of image-based social media platforms remains unexplored [19]. Systematic research is required to investigate how social media can be utilized to improve health communication.

As images are powerful tools of health communication [20], Instagram represents a unique social media platform that focuses on visual communication and the everyday ubiquity of smartphones. The use of betel nut in its various forms occurs throughout Southeast Asia and the Pacific Islands, where more than half of the world’s mobile subscribers live [21]. A recent study found that betel nut content is trending on Instagram, with evidence of increasing user engagement [22]. Therefore, it is important to explore how betel nut use is depicted on an image-based social media platform such as Instagram.

### Use of Social Media to Understand Betel Nut Use

Two theories that have been used to explain the uses and effects of social media include uses and gratifications (U&G theory) [23] and social cognitive theory (SCT) [24]. As originally conceived, the U&G theory suggests that individuals are driven to use media to satisfy certain social and psychological needs, such as, among others, wanting to integrate themselves into society and escape the real world [25]. Recent studies have identified factors that may motivate individuals to use Instagram, including wanting to interact with others, archiving or documenting one’s experiences, expressing oneself, escaping, and peeking or surveillance [23,26]. Such motives might also explain why individuals may choose to post betel nut–related content on Instagram.

On the other hand, SCT [27] posits that individuals can learn to adapt behaviors by modeling what they observe in the media. This assumption of SCT is consistent with the findings of recent studies, which indicate that as individuals are exposed to depictions of the use of a particular substance on social media, they become more likely to use the substance [28-30]. Using SCT as a framework may help understand how substance use behaviors are promoted on social media platforms [31]. Similarly, this study seeks to examine distinctions of betel nut–related content on Instagram to see how the social media platform may be used to promote betel nut use.

With 800 million users and 500 million users using the site daily, Instagram is the leading photo-sharing social media platform [32]. The majority (71%) of Instagram users are aged between 18 and 24 years and visit Instagram daily, with 55% using the platform several times a day [33]. As a networked visual social media platform, Instagram’s images, videos, and interactive content engagement can have a notable impact on people’s knowledge, attitudes, and perceptions of betel nut use. As Instagram is primarily a smartphone app, it is a technology that takes advantage of the portability and habitual use of mobile media [34]. As a result, the omnipresent nature of smartphones could offer a candid examination of betel nut practices within the everyday lives of its users. Furthermore, Instagram images and videos are uploaded and shared directly from a smartphone where other users interact with the content through likes and comments. The power of Instagram likes and comments has health implications, particularly among teenagers and young adults [35,36].

### Depicting Substance Use on Instagram

Instagram and similar image-sharing platforms have been the subject of several content analyses related to the promotion and use of addictive substances. For the most part, these studies favored visual over textual content utilizing topical hashtags to capture and document the context of individual use and marketing tactics undertaken on the platforms.

As a plant-based carcinogen, betel nut and betel quid have certain qualities that are similar to the marijuana plant. Instagram posts in marijuana-related hashtags showcased visual images of the marijuana plant [37]. Popular images included marijuana in its traditional forms (ie, buds and leaves) and nontraditional forms (ie, marijuana concentrates and marijuana-infused edibles). In addition, evidence of individuals using marijuana as well as marijuana-related advertisements demonstrated that marijuana content is prevalent on Instagram and helps to normalize and promote its use.

There is also evidence for the presence of hookah (water pipe) tobacco smoking content on visual social media platforms. Tumblr features prominent images of hookah pipes and smoke [38]. To a lesser degree, Tumblr also depicts the presence of waterpipe-related items such as hookah tobacco flavors, electronic hookahs, marijuana, alcohol, and tobacco products. Evidence of social gatherings, particularly between men and women, was also observed. Similarly, Instagram content using the hashtag *#hookah* found images featuring individual waterpipe use, paraphernalia, and social gathering [39]. The presence of promotional material was prominent on Instagram and often cross-promoted alcohol use. In this regard, the marketing power of Instagram [40] may be well-suited to promote waterpipe use and nightlife entertainment.

Recent studies have also documented electronic cigarettes (e-cigarettes) and vaping content on Instagram. In a dataset of 2208 Instagram images, the 3 most prominent themes were advertisements, products, and activity [41]. Advertisement themes explicitly promoted a commercial product and demonstrated the power of Instagram as a marketing tool. Activity themes showcased individuals exhaling aerosols, whereas product themes featured e-cigarettes or electronic juice (e-juice) bottles. An important finding is that likes and comments were found more with activity and product themes than with advertisement-themed images. Therefore, interactivity may prioritize particular Instagram content to larger audiences through social engagement on the platform [42]. A similar study considered e-cigarette visual content on both Instagram and Pinterest. Examining 1800 images from both image-based social media platforms, the popularity of marketing was evident, with 60% of Instagram posts devoted to marketing e-cigarettes. The second most common theme was customization, where users shared information about modifying e-cigarette devices for both functional and aesthetic purposes [43]. Product-related themes (ie, e-juice or flavors) were also evident. As Instagram is a very popular social media platform among teenagers, there is particular concern that Instagram postings may be especially attractive to youth, and that interest in flavors and juices will lure youth and others to try e-cigarettes.

Other health behaviors have been the subject of content analysis on Instagram. For example, a recent study examined the visual content of cigars and cigarillos on Instagram [44]. The findings indicated that Instagram promotes marijuana and tobacco use and promotion. In particular, Swisher products are used to promote the use of marijuana through blunt-making. Instagram images depicted individuals smoking little cigars and cigarillos as well as joints and blunts, though this was a less popular theme.

### Social and Cultural Characteristics of Betel Nut in the Western Pacific

Chamorros inhabited the Mariana Islands, an archipelago located in the Western Pacific, when European explorers first arrived in 1521. The Chamorro word for betel nut is *pugua*. Although betel nut is known for its lexical diversity [5], it is the preferred term for betel nut among the various ethnicities of Guam. There are several ways to prepare betel nut for consumption, but 2 distinct practices have emerged in Guam and Micronesia. Betel nut may be chewed with slaked lime (*afok*) and betel pepper leaf (*pupulu*) in the form of a betel quid [45]. This is the most common and traditional preparation throughout the world [46]. Peoples across Micronesia consume betel nut (typically unripened) in this way and spit out the juices as well as masticated quids [47]. However, most Chamorro chewers prefer mature nuts without lime and betel pepper leaves. Moreover, they ingest the juices and the betel nut itself [47]. In fact, Guam is the only island in Micronesia where people chew the mature areca nut [7].

Social and cultural motivations are prominent in the use of betel nut and prove difficult for sustaining health interventions [48,49]. *Pugua* represents Guam’s social glue, the bond that builds and maintains social networks among Chamorros and Micronesians [50]. It is shared among friends and family at weddings, anniversaries, and countless other social gatherings. Chewing betel nut is a habit that many Chamorros and Micronesians actively pass on to the next generation because it is a part of their heritage and is viewed as an important cultural identifier [47,51]. As a result, youth are continually exposed to betel nut practices leading to early onset of oral pathologies [46].

### Health and the Native Hawaiian and Pacific Islander Populations

As an image-based social media platform with health implications, Instagram intersects with 3 important concerns for the Native Hawaiian and Pacific Islander (NHPI) population. In comparison with other groups, the NHPI population is young and increasing at a higher rate [52]. As teens and young adults increasingly move toward visual social media platforms [53-55], image-based platforms represent an important space for public health interventions that speak to the changing demographics in NHPI communities. Furthermore, adolescents and young adults consume betel nut, and social pressure is an important contributing factor for deciding to chew [56]. Second, few data are available for NHPI on health and wellness; however, there is much need to address inequalities in vital areas of NHPI community life [57]. All too often, NHPI population data are aggregated with other Asian American groups [52]. Finally, the NHPI population faces challenges in maintaining their unique languages and cultures. Previous research has shown that Pacific Islander communities value social media as a way to preserve culture and indigenous knowledge [58,59]. Betel nut use is closely tied to culture in Guam [47,51,60,61] and throughout the USAPI [62,63]. All these denote important aspects for exploring how social media can promote health and behavioral change [64].

### The Goal of the Study

This study is an attempt to explore the ways in which betel nut is depicted on the photo-sharing site Instagram. As an image-based social media platform, little is known about what betel nut content is depicted on Instagram. This leads to the first research question:

- Research Question 1: How is betel nut portrayed and represented on Instagram in Micronesia?

Instagram interactivity through user content may shape user perceptions and choices undertaken on the site. For this study, social media engagement takes the form of *liking* and *commenting*. Thus, the second research question is as follows:

- Research Question 2: How do Instagram users engage with Instagram’s betel nut images and videos they encounter?

## Methods

### Data Collection and Analysis

**Figure 1:**
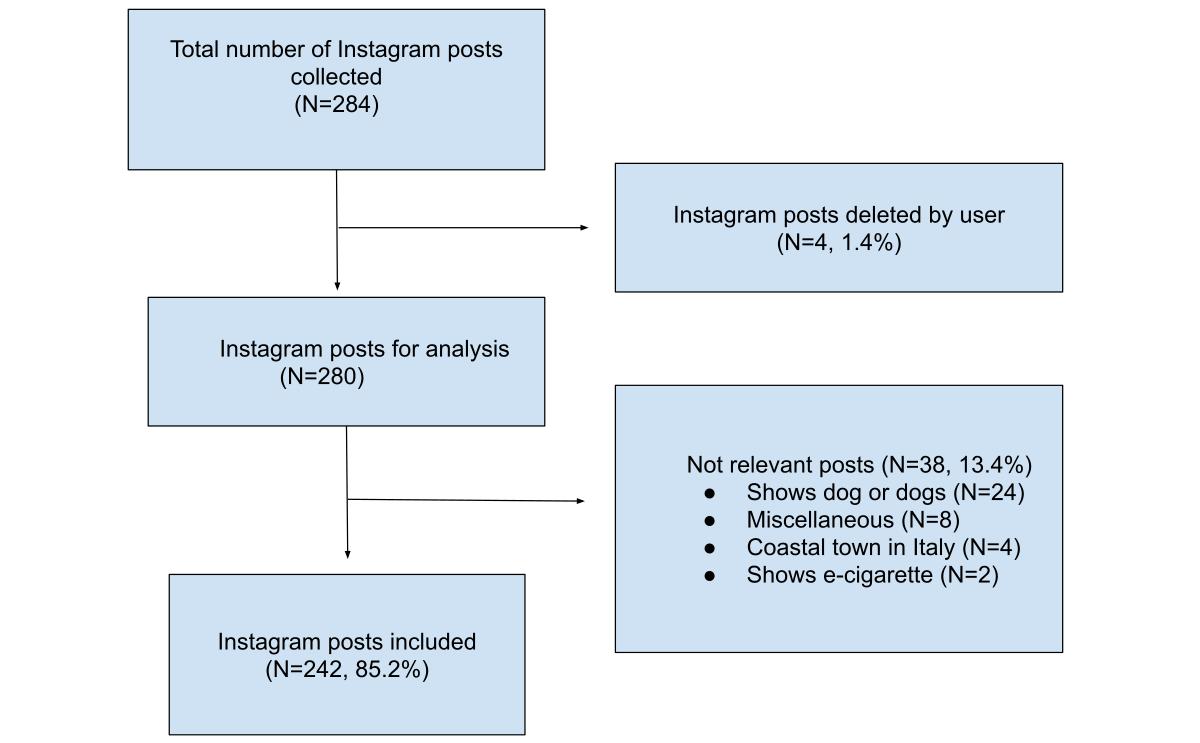
Inclusion of Instagram #pugua posts and reasons for exclusion. E-cigarette: electronic cigarette.

To acquire Instagram content, Netlytic was employed as a cloud-based social media analytics tool [65]. Netlytic has been used to collect, analyze, and visualize social media data on a variety of research topics from political discussion on Twitter [66] to health care community practice [67]. On May 22, 2017, public Instagram posts tagged *#pugua* were collected using the Instagram application programming interface (API) accessed through Netlytic. Data collection consisted of 284 Instagram posts dating back to 2011, uploaded by 180 unique Instagram users. A total of 4 posts were deleted from Instagram 1 month after data collection. All nonrelevant images and videos were also removed, resulting in a final dataset of 242 Instagram posts (234 images and 8 videos, 156 unique users). Figure 1 shows the number of posts identified and reasons for exclusion.

The unit of analysis was an Instagram post (metadata, image, caption, and comments). All images and videos were captured for data analysis. In addition, engagement was measured by adding the number of likes and comments for each post.

### Content Analysis

A coding scheme was constructed based on prior content analysis studies on substance use and Instagram and the existing literature on betel nut use [37,39,47]. Images and videos were coded in content categories that addressed the type of betel nut depicted in the image and any paraphernalia present. This provided the category *betel nut type* that concerned the depiction of betel nut tree or leaves, betel nut paraphernalia, and the type of betel nut. The codebook accounts for 2 distinct practices between Chamorro betel nut preparation (mature nut) and non-Chamorro, Micronesian (Chuukese, Palauan, and Yapese) preparation (unripe nut with slaked lime and/or tobacco). Depictions of betel nut use formed an additional category, though later removed in pilot testing. Similar to marijuana, betel nut provides the medium for products and accessories such as jewelry and other consumer goods. Betel nut product advertisements were coded.

Drawing on the strong link between betel nut and Chamorro culture [47], the codebook accounted for the visual and textual depiction of Chamorro culture. In addition, posts that referenced island life in Guam were considered a separate category. Working through the data, *stereotype* accounted for the contentious use of betel nut by Micronesians in Guam [60]. All 3 categories were placed under an umbrella theme of *cultural identifier*.

Prior studies have shown that betel nut use has notable social characteristics, such as encouraging social acceptance and social meetings [47,51,68]. For example, a bag of betel nut can promote future social acceptance among peers through respect for tradition and social promotion [51]. In addition, betel nut is often brought as a gift or favor for celebrations and social meetings [47]. Two content categories documented *social acceptance and promotion* and *social gathering*. Social gathering was further divided into 2 subthemes for either *celebration* or *social meeting*.

Betel nut practices intertwine with familial relations. Thus, children and youth are exposed to betel nut at an early age. The initial codebook accounted for the presence of youth (ie, children, adolescents, or young adults) in image or video content. The last content category referenced traditional or independent media coverage of betel nut use.

For coding, images and videos can be placed in multiple content categories. For example, if an image depicted the betel nut tree ripe with betel nut, the image was coded to both *betel nut tree or leaves* and *Yapese style* (if Yapese-style betel nut was shown in the tree). If the image also included the presence of young children, the image would receive an additional code for *children* under *youth presence*.

To judge the effectiveness of the codebook, 2 student coders assessed a sample (n=60) of the images and videos. A codebook with instructions and examples was created. After the first round of testing, codebook deficiencies led to improved content category definitions and the elimination of inadequate categorizations. For example, the depiction of betel nut use was difficult to detect in Instagram posts as it involves closed-mouth chewing. In addition, there were few perceived instances of betel nut chewing in the dataset, which were subsequently removed from the codebook.

A second round of interrater agreement was undertaken with a different set of more experienced coders. Utilizing their feedback on the coding instrument, a revised codebook was created, eliminating some content categories and improving the clarification of existing content categories. In all, 3 agreement measures demonstrated codebook validity: percentage concordance, Kappa α, and Krippendorff α. Given the multiple content categories and exploratory nature of the study, codebook concepts were considered reliable if percent agreement was ≥85%, and reliability coefficients were 0.50 or higher. A total of 6 primary content categories produced favorable levels of interrater agreement (Multimedia Appendix 1). Subthemes found in the betel nut type, cultural identifier, and youth presence categories yielded acceptable levels of interrater agreement ranging from 0.57 (betel nut tree or leaves, 85% agreement) to 1.00 (cultural stereotype, 100% agreement).

## Results

### Content Analysis

To answer the first research question, the 242 Instagram posts produced 386 top-level codebook references using NVivo 11. Of these, *betel nut type* was the most frequent primary content category, yielding 209 references or 54.1% (209/386) of the coded content. As shown in Table 1, betel palm tree or leaves and Chamorro, or Yapese-style betel nut, represented the majority of content references (182/209, 87.1%). Many of the Chamorro (56/64, 88%) or Yapese-style (34/48, 71%) betel nut were in a prepared state ready for consumption. Betel nut paraphernalia (mainly betel nut cutters) was displayed in 12.4% (26/209) of the referenced category content.

**Table 1:** Distribution of betel nut type categories (N=209).

Theme	References, n (%)^a^
Betel nut tree or leaves	70 (33.5)
Chamorro style nut	64 (30.6)
If Chamorro style nut, is it prepared?	56 (26.8)
Yapese style nut	48 (23.0)
If Yapese style nut, is it prepared?	34 (16.3)
Betel nut paraphernalia	26 (12.4)

^a^Percentages do not total to 100% because of the two conditional categories with their listed percentages.

The second most frequent content referenced on #pugua was images and videos addressing various aspects of cultural identification. Cultural identification references accounted for 22.3% (86/386) of the coded data (Table 2). Of these, posts referencing visual and/or textual aspects of Chamorro culture represented more than half (55/86, 64%) of the content category. A total of 23% (20/86) of cultural content displayed elements of island life in Guam. As spitting has become a sign of racial tension on the island, the remaining content (12/86, 14%) depicted stereotypical images of Micronesian use of betel nut. This included images of red spittle on the ground or public *no spitting* signs, which serve as blatant racialized markers of tension between Chamorros and Micronesians.

**Table 2:** Distribution of cultural identification categories (N=86).

Theme	References, n (%)
Chamorro culture	55 (64)
Island life	20 (23)
Stereotype	12 (14)

Social acceptance and promotion comprised approximately 10.1% (39/386) of the dataset. As shown in Table 3, social acceptance and promotion included a bag or bags of betel nut. Youth presence (N=24) and betel nut product advertising (N=23) represented 6% of the overall content. Youth presence was equally split between children (aged 0-10 years) and adolescents (aged 11-17 years). Some Instagram posts showed children or adolescents handling betel nut in a playful manner. A total of 23 image or video references attempted to use #pugua to sell or market betel nut products, clothing, or accessories. A total of 5 #pugua Instagram posts depicted media coverage referencing documentaries addressing health concerns or the practice of betel nut chewing.

**Table 3:** Distribution of social acceptance and promotion, youth presence, betel nut products and accessories, and media coverage categories (N=386).

Theme	References, n (%)
Social acceptance and promotion	39 (10.1)
Youth presence	24 (6.2)
Betel nut products and accessories	23 (6.0)
Media coverage	5 (1.3)

### Social Engagement in #pugua

**Figure 2:**
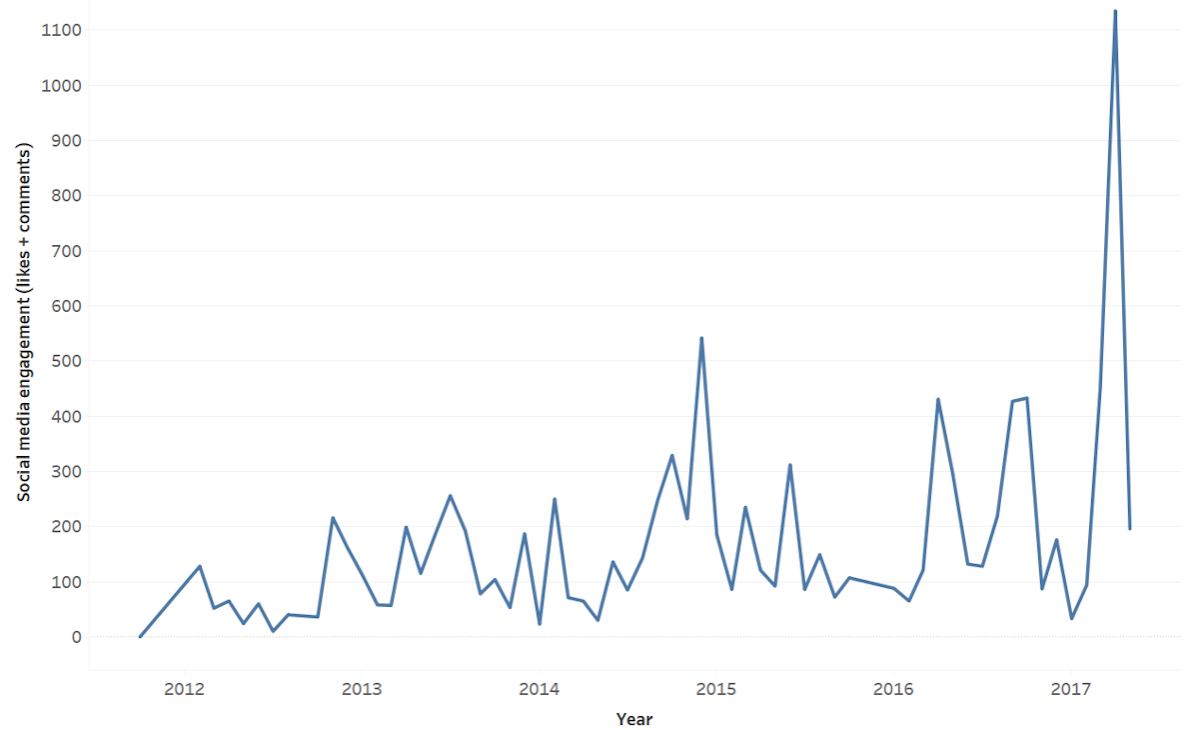
Social media engagement on #pugua (N=242).

The average engagement score (likes + comments) was 43.33 (SD 48.47) and displayed an upward trend (Figure 2).

To determine if particular #pugua content related to social media engagement, engagement scores were classified into 3 categories: low (0-18), medium (19-49), and high (≥50). Matrix coding resulted in 68 low engagement posts, 111 medium engagement posts, and 62 high engagement posts. For each engagement group, we ranked the 3 most frequent categories (Table 4).

**Table 4:** Top 3 content categories by level of engagement.

Ranking	Low	Medium	High
1	Chamorro-style betel nut (22)^a^	Betel nut tree or leaves (35)	Chamorro culture (18)
2	Betel nut tree or leaves (20)	Chamorro culture (30)	Chamorro-style betel nut (16)
3	Yapese-style betel nut (17)	Chamorro-style betel nut (27)	Betel nut tree or leaves (15)

^a^Numbers in parentheses indicate the frequency of the content category appearing in the matrix cell.

**Figure 3:**
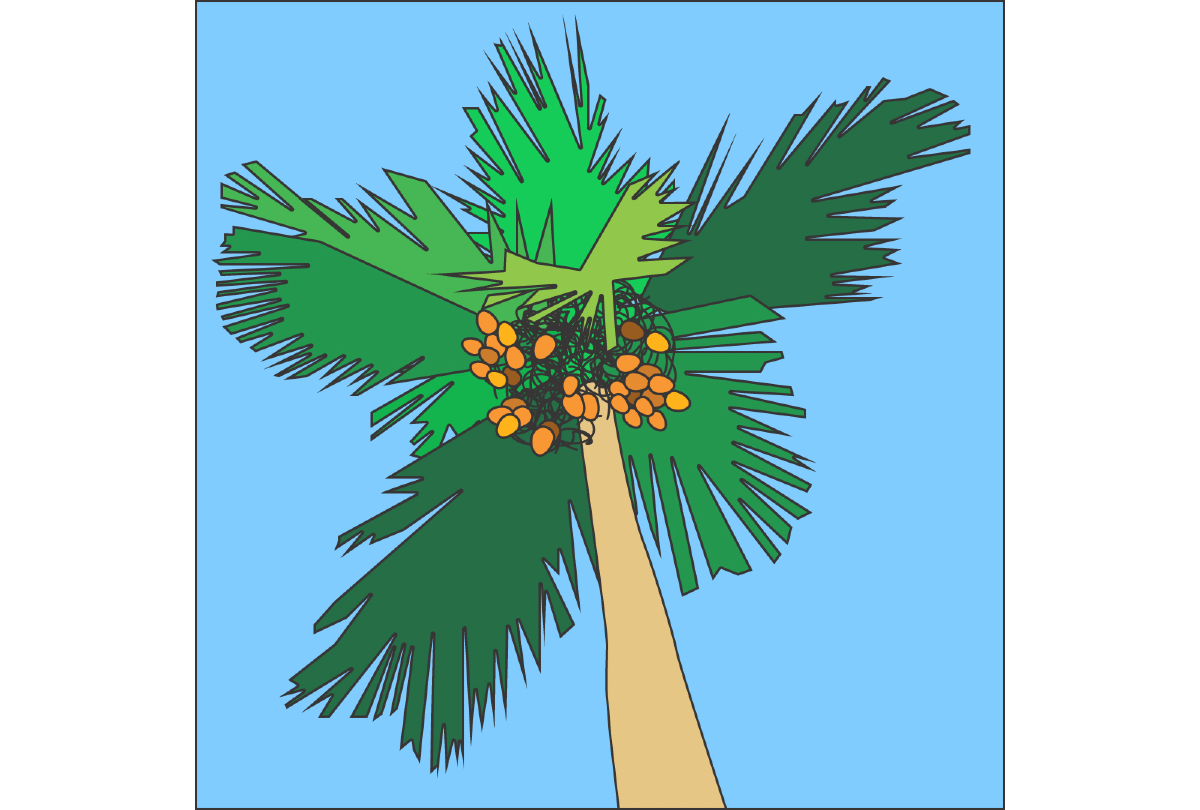
Artist’s impression of a typical high engagement #pugua image featuring a betel palm tree and mature betel nut. Illustration by Jessica K Sato.

**Figure 4:**
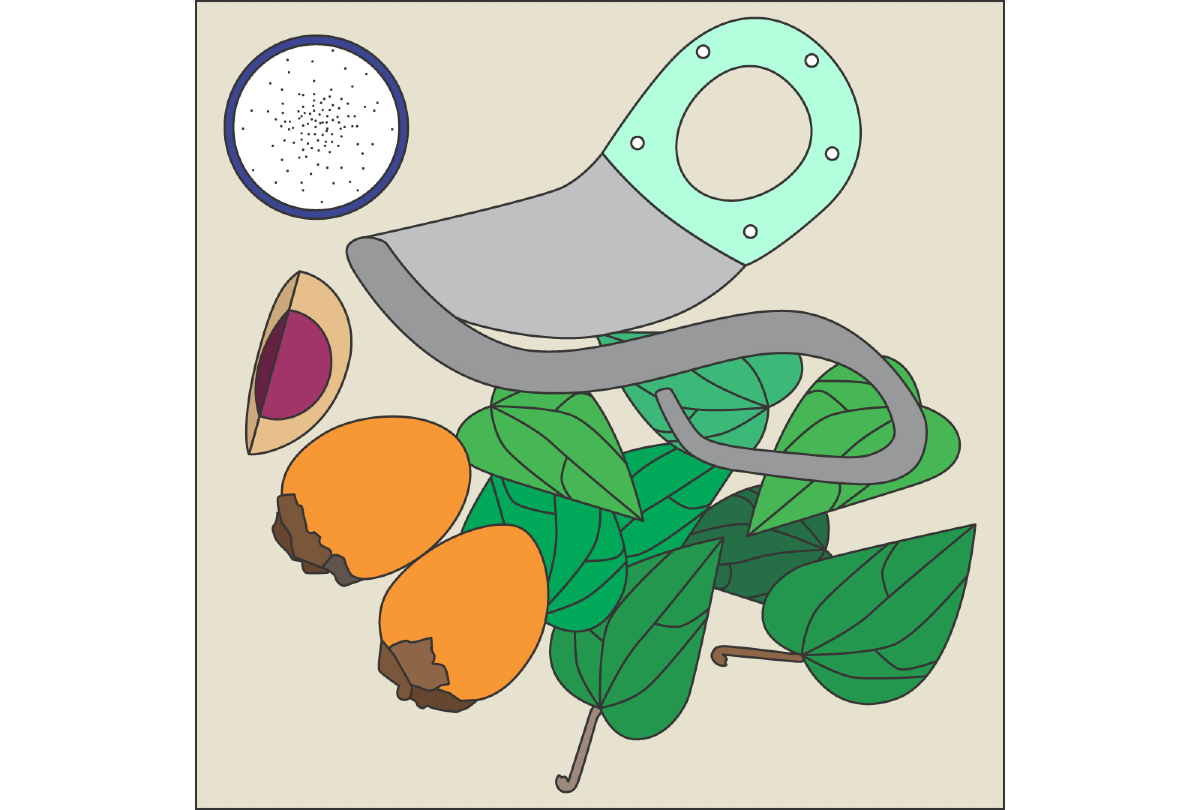
Artist’s impression of a typical high engagement #pugua image featuring mature betel nut, betel pepper leaves, slaked lime, and betel nut cutter. Illustration by Jessica K Sato.

For the second research question, Instagram posts featuring betel nut tree or leaves (Figure 3) and Chamorro-style betel nut (Figure 4) consistently appear regardless of the level of social engagement. When Chamorro-style betel nut is shown, it is often in a prepared state. At the medium and high engagement level, 95% (41/43) of the Chamorro-style betel nut posts showed betel nut in a Chamorro-prepared style (diced or cut). For low engagement, 68% (15/22) of Chamorro-style betel nut posts were in a prepared state (8/17, 47% for Yapese-style betel nut). For Instagram posts that generated medium and high engagement levels, visual or textual aspects of Chamorro culture were notably present. Highly engaged #pugua posts were more likely to feature Chamorro betel nut preparation or culture compared with other content categories.

## Discussion

### Principal Findings

As shown in previous studies, betel nut use and practice vary by geographic region [69,70], and Guam is a unique case [47,51,68]. For Guam, betel nut chewing practices that are exhibited offline may replicate themselves on the web within the pugua hashtag. Prior evidence indicated 2 distinct betel nut chewing practices in Guam: Chamorro style and Yapese style [47]. Content analysis confirmed these 2 distinct preparation practices by noting the type of betel nut displayed (mature or unripe), as well as the distinct preparation practices before consumption. The Chamorro style prepares a mature betel nut that is often diced before consumption. In contrast, the Yapese style reflects Micronesian cultural preferences to consume an unripe nut with a piper betel leaf mixed with slaked lime or tobacco to form a betel quid. Aside from Instagram posts that featured betel nut tree or leaves, Chamorro- and Yapese-style betel nut and their respective preparation states were the most frequently occurring content in #pugua. In addition, approximately 10% of Instagram posts showed betel nut cutters that slice betel nut for consumption. As a result, it is plausible that #pugua users on Instagram have opportunities to observe how the betel nut moves from a whole nut to a chewable form. Further studies could determine if these visual depictions of betel nut cultural practices lead to greater uptake in betel nut use, especially among adolescents and young adults.

Approximately 1 out of every 5 #pugua posts referenced Chamorro cultural content either through image/video, text, or both. Pugua has been referenced as the social glue for Chamorro culture. In the exploration of #pugua, there were depictions of Chamorro culture such as dance, art, and festivals without the display of betel nut in the Instagram content. As prior work has demonstrated that Chamorros use social media to learn, capture, and preserve their culture [59], public health interventions should carefully consider how their visual messages link betel nut content on Instagram to Chamorro cultural identification.

### #pugua and Social Engagement

As Instagram is a visual social media platform, the circulation of the image through likes and comments may have more value than the image content [71]. The findings from this study determined that certain Instagram #pugua posts had higher social engagement than others. Instagram #pugua posts with moderate or high amounts of social engagement centered around visual content privileging Chamorro culture, Chamorro-style betel nut, and betel nut tree and leaves. Instagram and the smartphone favor *in the moment* sharing often reflecting everyday life instances. As betel nut use is an underexamined research phenomenon, there is a need to understand who, where, when, how, and why people chew betel nut [72]. The exploratory results of this study provide insights into the who, how, and why people in Guam and Micronesia chew betel nut and confirm the existing limited research in this area. Thus, this study represents a first step toward understanding how image-based social media platforms capture and collate information on betel nut practices.

### Comparison With Prior Work

As a plant-based carcinogen, the areca nut and betel pepper leaf share certain qualities with the marijuana plant. These psychoactive plants provide facilities for ingesting and supplementing the use of the product. For example, betel nut may be consumed within the betel pepper leaf. Similarly, marijuana buds or leaves may be consumed or form additional use practices. Both plant-based products supply the medium for additional products and accessories. As a result, betel nut practice and use on Instagram may closely resemble the practice and use of marijuana on Instagram. Comparing the study’s findings with the large-scale work on marijuana and Instagram [37], there are some similarities, but also notable differences.

As indicated by previous research on marijuana and Instagram [37], the most frequent Instagram posts involved showing the betel palm tree and leaves, mature or unripe betel nut, and its various consumption practices. In this regard, the betel nut and marijuana plant share certain visual characteristics that appeal to their respective audiences on Instagram. Therefore, public health professionals should consider how to balance appealing visual content with helpful information for young people about the known risks of betel nut use.

The consumption and masticatory use of betel nut did not appear to be compelling content on Instagram. Very few Instagram posts featured chewing of betel nut or betel quid. If it was displayed, it was only through a closed mouth where a lump could be witnessed in the person’s jaw. Previous studies have shown that betel nut chewing and spitting are bad habits that undermine public health [73]. On a visual communication medium such as Instagram, it is difficult to portray betel nut chewing as novel and interesting to facilitate social networking. This finding is in contrast to the work done on the use of marijuana in social media [37,74]. Previous research has shown that marijuana use on social media facilitates social networking about marijuana, particularly among young adults and underage youth. Ingesting marijuana in its traditional plant-based and novel forms (edibles and concentrates) is perhaps more visually appealing and also helps to normalize and promote marijuana use as a form of practice. Similarly, e-cigarette, hookah, and cigar/cigarillos Instagram content featured activity and use themes such as smoking, exhaling clouds, and smoke tricks [38,39,41,43,44]. The exploratory findings in this study did not observe the use of betel nut on an image-based social media platform.

Advertising and marketing tactics have been well documented for e-cigarette and marijuana use on Instagram [30,37,41]. However, there was little evidence of advertising and product promotion of betel nut in #pugua. This is most likely a reflection of the lack of a clear marketplace that sells specific accessories and products related to its consumption, such as vape shops and marijuana dispensaries. Betel nut products that were advertised tended toward clothing or jewelry accessories made from betel palm tree or pepper leaf fibers. These Instagram posts are often linked to specific references to Chamorro culture. Therefore, it is plausible that #pugua on Instagram functions less as a marketplace and more as a cultural space.

### Limitations

There are limitations to this study. The dataset was small and limited the generalizability of the study findings. A paid Netlytic account captured all posts that met the inclusion criteria. Given the size of the dataset, all #pugua posts were analyzed, and there was no sampling required. However, data collection and sampling remain a challenge for capturing image-based social media content because of API restrictions and privacy concerns [19]. Algorithms on Instagram also influence the visibility of the #pugua content [75] and certainly contribute toward its social engagement. Nonetheless, caution should be exercised to avoid generalizing the findings of #pugua to all Instagram users and contexts. Future work should empirically test the study’s implications through experiments documenting image content effects on Instagram betel nut users or pursue large-scale data collection of #betelnut and regional betel nut terms as hashtags on social media platforms.

### Conclusions

This study analyzed the ways in which betel nut is depicted on the photo-sharing site Instagram. A content analysis of #pugua explored betel nut practices in #pugua and its engagement on the platform. The study findings suggest that betel nut preparation practices reflect offline practices in Guam and Micronesia. In addition, socially engaged content is more likely to reflect the areca nut, betel palm tree or leaves and Chamorro cultural sensibilities. Compared with prior work on substance use and Instagram, #pugua content demonstrated similarities to marijuana in terms of depicting the betel palm tree, areca nut, and quid ingredients. In addition, advertisements of betel nut clothing and accessories were observed. In contrast to prior work, there was little evidence of actual betel nut use and consumption, which is typically a prominent feature in marijuana, hookah, and vaping content on image-based social media.

The findings from this study emphasize the cultural relevance of betel nut use in Micronesia. The findings provide a basis for empirically testing hypotheses related to the etiological roles of cultural identity and pride in shaping betel nut use behavior among Micronesians, particularly youths and young adults. Such research is likely to inform the development of culturally relevant betel nut prevention and cessation programs.

## Appendix

Multimedia Appendix 1 Reliability indicators for codebook categories.

## Abbreviations

API: application programming interface
e-cigarettes: electronic cigarettes
e-juice: electronic juice
NIH: National Institutes of Health
NHPI: Native Hawaiian and Pacific Islanders
SCT: social cognitive theory
U&G: uses and gratifications
USAPI: US-Affiliated Pacific Islands
