# Supplementary material for: An Instagram-Based Study to Understand Betel Nut Use Culture in Micronesia: Exploratory Content Analysis
Source: J Med Internet Res. 2020 Jul 9;22(7):e13954. doi: 10.2196/13954 (PMC7381034; doi:10.2196/13954)
Supplement: Multimedia Appendix 1 [file jmir_v22i7e13954_app1.docx]

**Table A1. Reliability indicators for codebook categories.**

| **Theme** | | **Definition** | **%** | **κ** | **α** |
| --- | --- | --- | --- | --- | --- |
|  | |  |  |  |  |
| **Betel nut type** | | Images or video feature betel nut tree or leaves, betel nut, and/or betel nut paraphernalia. Theme is further divided by the following subthemes: tree or leaves, Chamorro style, Chamorro preparation, Yapese style, Yapese preparation and betel nut paraphernalia. | 97 | 0.921 | 0.921 |
|  | Betel nut tree or leaves | Image or video displays betel nut tree or leaves | 87 | 0.590 | 0.587 |
|  | Chamorro style betel nut | Image or video shows mature betel nut with red, yellow, or orange coloring | 92 | 0.805 | 0.806 |
|  | Prepared Chamorro style betel nut | If a Chamorro style nut is displayed, then is it shown in a prepared state? Betel nut should be diced or cut. | 93 | 0.792 | 0.793 |
|  | Yapese style betel nut | Image or video shows betel nut as a green, immature nut | 93 | 0.792 | 0.793 |
|  | Prepared Yapese style betel nut | If a Yapese style nut is displayed, then is it shown with preparation materials such as slaked lime and/or tobacco. Lime and tobacco are associated with Yapese betel nut preparation | 98 | 0.900 | 0.901 |
|  | Betel nut paraphernalia | Image or video displays betel nut cutters or other instruments to facilitate betel nut preparation or use | 97 | 0.869 | 0.870 |
| **Cultural identifier** | | Instagram content portrays a Chamorro cultural identity, island life or South Pacific stereotypes. Subthemes are Chamorro culture, island life or stereotype. | 97 | 0.889 | 0.890 |
|  | Chamorro culture | Image, video and/or text displays Chamorro cultural identity. Includes visual or textual reference to Chamorro food, dance or customs. | 97 | 0.733 | 0.734 |
|  | Stereotype | Image or video portrays cultural stereotypes from Micronesia or South Pacific. Includes 'no spitting' signs/comments | 100 | 1.00 | 1.00 |
|  | Island life | Image, video and text depicts island lifestyle in Guam. References used are beaches, scenic settings, geography, etc. | 100 | 1.00 | 1.00 |
| **Social acceptance and promotion** | | Image or video displays a bag or bags of betel nut. | 98 | 0.938 | 0.938 |
| **Accessories, products or advertisements** | | Image or video features an advertisement or attempt to sell betel nut-related accessories such as jewelry, earrings or clothes | 95 | 0.700 | 0.702 |
| **Youth Presence** | | Image or video shows the presence of youth. Subthemes are infant, toddler or young child (0-10yrs) or adolescent (11-17yrs) | 97 | 0.782 | 0.784 |
|  | Children | Image or video shows an infant, toddler, or young child between infant and 10 years old. | 98 | 0.792 | 0.793 |
|  | Adolescent | Image or video shows adolescent(s) between 11-17 years old. | 98 | 0.792 | 0.793 |
| **Media Coverage** | | Image or video reference any media coverage (documentaries or news stories) about betel nut | 100 | 1.00 | 1.00 |
